# Supplementary figures and images for: T-follicular helper cells are epigenetically poised to transdifferentiate into T-regulatory type 1 cells
Source: eLife. 2024 Nov 22;13:RP97665. doi: 10.7554/eLife.97665 (PMC11584177; doi:10.7554/eLife.97665)

Fig. 6

A

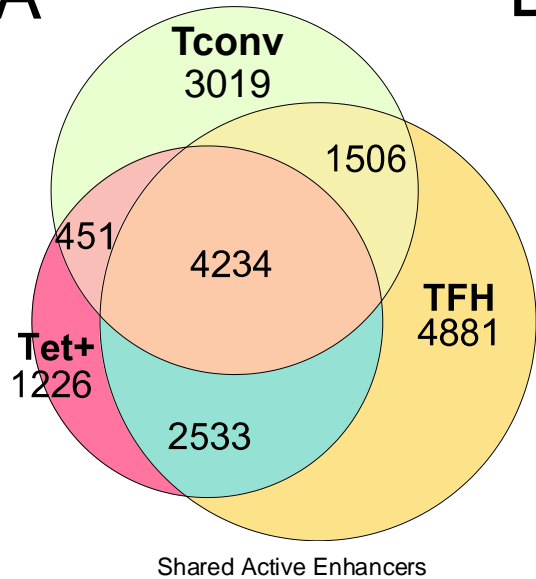

B

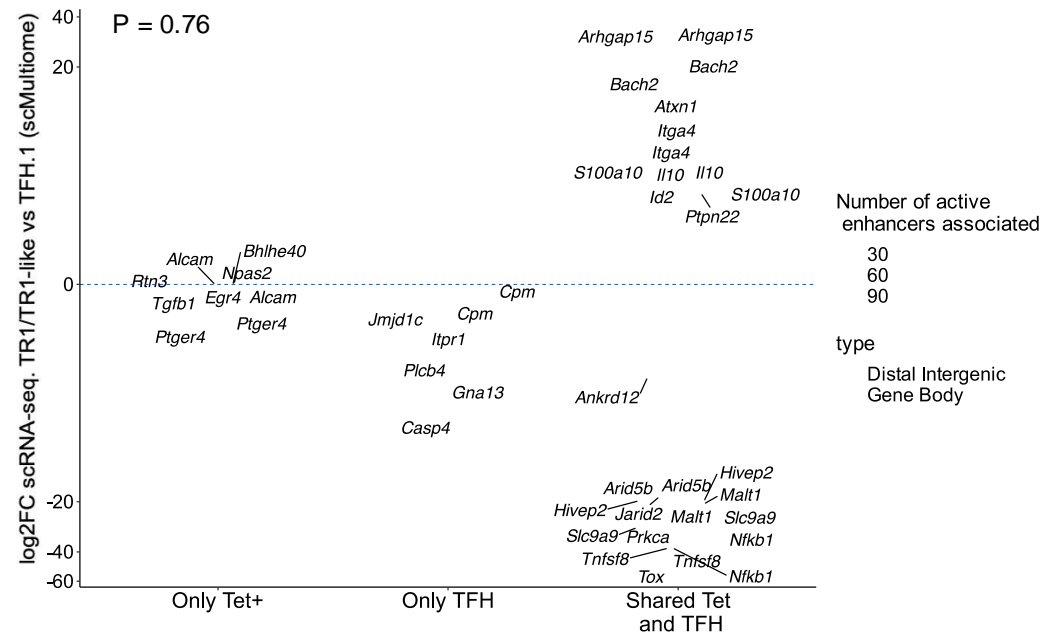

C

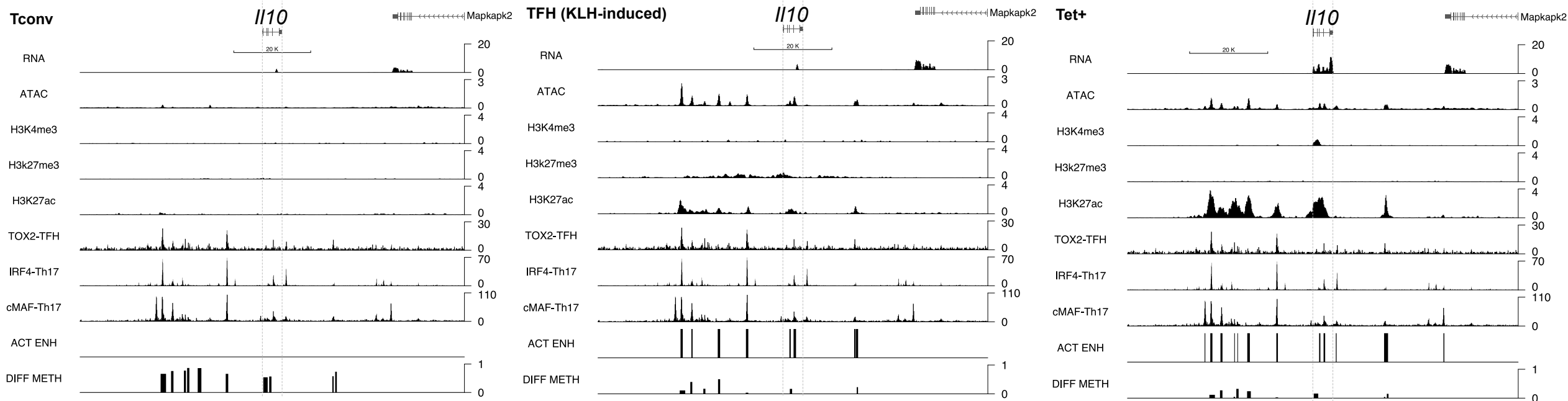

Supplement: Figure 4—source data 1. [file elife-97665-fig4-data1.pdf]
