# Supplementary material for: T-follicular helper cells are epigenetically poised to transdifferentiate into T-regulatory type 1 cells
Source: eLife. 2024 Nov 22;13:RP97665. doi: 10.7554/eLife.97665 (PMC11584177; doi:10.7554/eLife.97665)
Supplement: Supplementary file 1. [file elife-97665-supp1.docx]

**Supplementary Table 1**

| Gene type | Genes (protein) | Described in  TR1 (ref) | Described in other  Treg types (ref) | Described in  TFH (ref) | DE in Tet+  (vs. TFH) |
| --- | --- | --- | --- | --- | --- |
| Cell adhesion molecules | Cd226 | + (1) | ND | ND | +++ |
|  | Itga2 (CD49b) | + (1) | ND | ND | ++ |
|  | Itgae (CD103) | + (2) | + (3) | ND | +++ |
|  | Ocln | ND | ND | ND | +++ |
|  | S1pr2 | ND | ND | + (4) | - |
|  | Sell (CD62L) | - (5) | + (6) | - (7) | +++ |
|  | Selplg (Psgl1) | ND | ND | - (8) | +++ |
| Chemokyne receptors | Ccr5 | + (9) | + (10) | + (11) | +++ |
|  | Ccr7 | ND | + (12) | - (13) | +++ |
|  | Cxcr3 | + (14) | + (12) | In some subsets (15) | +++ |
|  | Cxcr4 | ND | ND | + (16) | NS |
|  | Cxcr5 | ND | ND | + (17) | - - |
| Co-estimulatory molecules | Cd28 | + (18) | ND | + (19) | + |
|  | Cd40lg | ND | ND | + (20) | + |
|  | Icos | + (21) | + (22) | + (23) | - |
|  | Klrk1 (NKG2D) | ND | + (24) | ND | NS |
|  | Sh2d1a (SAP) | ND | ND | + (25) | - - |
|  | Tnfrsf4 (Ox40) | + (26) | + (27) | + (28) | ++ |
|  | Tnfrsf18 (GITR) | + (18) | + (29) | ND | + |
|  | Tnfsf4 (Ox40L) | ND | ND | ND | +++ |
| Co-inhibitory molecules | Ctla4 | + (30) | + (31) | + (32) | ++ |
|  | Fasl | ND | + (33) | ND | ++ |
|  | Havcr2 (TIM-3) | + (34) | + (35) | + (36) | +++ |
|  | Lag3 | + (1) | + (37) | ND | +++ |
|  | Pdcd1 (PD-1) | + (9) | + (38) | + (17) | - - |
|  | Tigit | + (39) | + (40) | + (41) | NS |
| Cytokines | Ebi3 (IL27b) | ND | + (42) | ND | + |
|  | Ifng | + (43) | ND | ND | +++ |
|  | Il10 | + (44) | + (45) | + (46) | +++ |
|  | Il21 | + (47) | ND | + (48) | NS |
|  | Il4 | - (44) | ND | + (49) | - |
|  | Il5 | + (44) | ND | ND | NS |
|  | Mcub (Areg) | + | + (50) | ND | NS |
|  | Tgfb1 | + (44) | + (45) | ND | NS |
| Cytokine receptors | Il10ra | + (51) | + (52) | ND | ++ |
|  | Il12rb2 | ND | + (53) | ND | - - |
|  | Il21r | + (47) | + (54) | + (48) | - |
|  | Il27ra | + (55) | + (56) | + (57) | + |
|  | IL7r (CD127) | - (21) | - (58) | - (59) | +++ |
|  | IL17rc | ND | ND | ND | NS |
|  | Il2ra (CD25) | - (18) | + (60) | - (61) | +++ |
|  | Tgfbr1 | - (51) | + (62) | ND | - |
|  | Tgfbr2 | - (51) | + (63) | ND | NS |
|  | Tgfbr3 | - (51) | ND | ND | ++ |
|  | Atf6 | - (51) | ND | ND | NS |
|  | Ahr | + (64) | ND | ND | +++ |
|  | Ajuba | ND | ND | ND | +++ |
|  | Ascl2 | ND | ND | + (65) | - |
|  | Bach2 | - (66) | + (67) | - (68) | ++ |
|  | Batf | + (69) | ND | + (70) | - |
|  | Bcl6 | ND | ND | + (71) | - - |
|  | Bhlhe40 | + (51) | ND | ND | ++ |
|  | Bmyc | - (51) | ND | ND | NS |
|  | Cbfa2t3 | ND | ND | ND | - |
|  | Cebpa | ND | ND | + (72) | - - |

| Transcription factors | Dbp | - (51) | ND | ND | NS |
| --- | --- | --- | --- | --- | --- |
|  | E2f1 | + (51) | + (73) | ND | NS |
|  | Egr2 | + (74) | ND | ND | - |
|  | Elk4 | - (51) | ND | ND | - |
|  | Eomes | + (75) | + (76) | ND | ++ |
|  | FoxP1 | ND | ND | - (77) | + |
|  | FoxP3 | ND | + (78) | ND | - - |
|  | Grhl1 | ND | ND | ND | NS |
|  | Hmgb2 | + (51) | ND | ND | NS |
|  | Id2 | + (51) | + (79) | - (80) | ++ |
|  | Id3 | - (51) | + (79) | + (80) | - |
|  | Irf1 | + (69) | ND | ND | + |
|  | Irf4 | + (81) | + (3) | + (82) | ++ |
|  | Jdp2 | ND | ND | ND | - - |
|  | Klf2 | ND | ND | - (83) | +++ |
|  | Lef1 | ND | ND | + (84) | NS |
|  | Lilrb4a | ND | + (85) | ND | NS |
|  | Maf | + (47) | ND | + (86) | - |
|  | Myb | - (51) | + (87) | ND | NS |
|  | Mybl2 | + (51) | ND | ND | - - |
|  | Myc | - (51) | - (88) | ND | NS |
|  | Nfia | ND | ND | ND | - - |
|  | Nfil3 | + (34) | ND | ND | ++ |
|  | Nr1h3 (LXR𝛂) | + (51) | + (89) | ND | - |
|  | Pax5 | ND | ND | ND | - - |
|  | Pax9 | ND | ND | ND | ++ |
|  | Pou2af1 (OcaB) | ND | ND | ND | - |
|  | Prdm1 (Blimp-1) | + (90) | ND | + (91) | +++ |
|  | Rbpj | + (51) | + (92) | ND | + |
|  | Runx2 | + (51) | ND | ND | ++ |
|  | Rora | + (51) | ND | - (93) | - - |
|  | S1pr1 | ND | ND | - (83) | ++ |
|  | Six5 | ND | ND | ND | NS |
|  | Sox4 | - (51) | - (94) | ND | NS |
|  | Sox8 | ND | ND | ND | - - |
|  | Stat1 | + (95) | ND | + (96) | NS |
|  | Stat3 | + (97) | ND | + (23) | NS |
|  | Stat4 | ND | - (98) | + (99) | - |
|  | Tbx21 (T-bet) | + (75) | ND | - (84) | ++ |
|  | Tcf7 | ND | ND | + (84) | NS |
|  | Tox2 | ND | ND | + (80) | - - |
|  | Vdr | ND | + (100) | ND | NS |
|  | Zbtb16 (PLZF) | + (51) | ND | ND | NS |
| Secretion proteins | Chgb | ND | ND | + (101) | NS |
|  | Gzmb (Granzyme B) | + (102) | + (103) | ND | +++ |
| Enzymes | Cblb | ND | + (104) | ND | + |
|  | Entpd1 (CD39) | + (105) | + (106) | ND | +++ |
|  | Itk | + (107) | + (108) | ND | - |
|  | Nt5e (CD73) | + (105) | + (106) | + (109) | NS |
|  | Serpinb6b | + (51) | + (110) | ND | NS |
|  | Serpinb9 | + (51) | ND | ND | NS |

+++ FC>4

++ 4>FC>2

+ 2>FC>1

NS 1>FC>-1

- -1>FC>-2

- - -2>FC>-4

- - - FC<-4

ND Not determined

REFERENCES FOR SUPPLEMENTARY TABLE 1

1. Gagliani, N. *et al.* Coexpression of CD49b and LAG-3 identifies human and mouse T regulatory type 1 cells. *Nat. Med.* **19**, 739-746 (2013).

2. Duan, W., So, T., Mehta, A.K., Choi, H. & Croft, M. Inducible CD4+LAP+Foxp3- regulatory T cells suppress allergic inflammation. *J. Immunol.* **187**, 6499-6507 (2011).

3. Cretney, E. *et al.* The transcription factors Blimp-1 and IRF4 jointly control the differentiation and function of effector regulatory T cells. *Nat. Immunol.* **12**, 304-311 (2011).

4. Moriyama, S. *et al.* Sphingosine-1-phosphate receptor 2 is critical for follicular helper T cell retention in germinal centers. *J. Exp. Med.* **211**, 1297-1305 (2014).

5. Bollyky, P.L. *et al.* ECM components guide IL-10 producing regulatory T-cell (TR1) induction from effector memory T-cell precursors. *Proc. Natl. Acad. Sci. U. S. A.* **108**, 7938-7943 (2011).

6. Biswas, M., Kumar, S.R.P., Terhorst, C. & Herzog, R.W. Gene Therapy With Regulatory T Cells: A Beneficial Alliance. *Front. Immunol.* **9**, 554 (2018).

7. Fazilleau, N., McHeyzer-Williams, L.J., Rosen, H. & McHeyzer-Williams, M.G. The function of follicular helper T cells is regulated by the strength of T cell antigen receptor binding. *Nat. Immunol.* **10**, 375-384 (2009).

8. Poholek, A.C. *et al.* In vivo regulation of Bcl6 and T follicular helper cell development. *J. Immunol.* **185**, 313-326 (2010).

9. Alfen, J.S. *et al.* Intestinal IFN-gamma-producing type 1 regulatory T cells coexpress CCR5 and programmed cell death protein 1 and downregulate IL-10 in the inflamed guts of patients with inflammatory bowel disease. *J. Allergy Clin. Immunol.* **142**, 1537-1547 e1538 (2018).

10. Bystry, R.S., Aluvihare, V., Welch, K.A., Kallikourdis, M. & Betz, A.G. B cells and professional APCs recruit regulatory T cells via CCL4. *Nat. Immunol.* **2**, 1126-1132 (2001).

11. Miller, S.M. *et al.* Follicular Regulatory T Cells Are Highly Permissive to R5-Tropic HIV-1. *J. Virol.* **91** (2017).

12. Smigiel, K.S. *et al.* CCR7 provides localized access to IL-2 and defines homeostatically distinct regulatory T cell subsets. *J. Exp. Med.* **211**, 121-136 (2014).

13. Haynes, N.M. *et al.* Role of CXCR5 and CCR7 in follicular Th cell positioning and appearance of a programmed cell death gene-1high germinal center-associated subpopulation. *J. Immunol.* **179**, 5099-5108 (2007).

14. Kunicki, M.A., Amaya Hernandez, L.C., Davis, K.L., Bacchetta, R. & Roncarolo, M.G. Identity and Diversity of Human Peripheral Th and T Regulatory Cells Defined by Single-Cell Mass Cytometry. *J. Immunol.* **200**, 336-346 (2018).

15. Chevalier, N. Quantifying helper cell function of human TFH cells in vitro. *Methods Mol. Biol.* **1291**, 209-226 (2015).

16. Allen, C.D. *et al.* Germinal center dark and light zone organization is mediated by CXCR4 and CXCR5. *Nat. Immunol.* **5**, 943-952 (2004).

17. Crotty, S. Follicular helper CD4 T cells (TFH). *Annu. Rev. Immunol.* **29**, 621-663 (2011).

18. Zeng, H., Zhang, R., Jin, B. & Chen, L. Type 1 regulatory T cells: a new mechanism of peripheral immune tolerance. *Cell. Mol. Immunol.* **12**, 566-571 (2015).

19. Linterman, M.A. *et al.* CD28 expression is required after T cell priming for helper T cell responses and protective immunity to infection. *Elife* **3** (2014).

20. Vinuesa, C.G., Tangye, S.G., Moser, B. & Mackay, C.R. Follicular B helper T cells in antibody responses and autoimmunity. *Nat. Rev. Immunol.* **5**, 853-865 (2005).

21. Haringer, B., Lozza, L., Steckel, B. & Geginat, J. Identification and characterization of IL-10/IFN-gamma-producing effector-like T cells with regulatory function in human blood. *J. Exp. Med.* **206**, 1009-1017 (2009).

22. Strauss, L. *et al.* Expression of ICOS on human melanoma-infiltrating CD4+CD25highFoxp3+ T regulatory cells: implications and impact on tumor-mediated immune suppression. *J. Immunol.* **180**, 2967-2980 (2008).

23. Nurieva, R.I. *et al.* Generation of T follicular helper cells is mediated by interleukin-21 but independent of T helper 1, 2, or 17 cell lineages. *Immunity* **29**, 138-149 (2008).

24. Fujio, K., Okamura, T. & Yamamoto, K. The Family of IL-10-secreting CD4+ T cells. *Adv. Immunol.* **105**, 99-130 (2010).

25. Crotty, S., Kersh, E.N., Cannons, J., Schwartzberg, P.L. & Ahmed, R. SAP is required for generating long-term humoral immunity. *Nature* **421**, 282-287 (2003).

26. Ito, T. *et al.* OX40 ligand shuts down IL-10-producing regulatory T cells. *Proc. Natl. Acad. Sci. U. S. A.* **103**, 13138-13143 (2006).

27. Fontenot, J.D. *et al.* Regulatory T cell lineage specification by the forkhead transcription factor foxp3. *Immunity* **22**, 329-341 (2005).

28. Jacquemin, C. *et al.* OX40 Ligand Contributes to Human Lupus Pathogenesis by Promoting T Follicular Helper Response. *Immunity* **42**, 1159-1170 (2015).

29. Shimizu, J., Yamazaki, S., Takahashi, T., Ishida, Y. & Sakaguchi, S. Stimulation of CD25(+)CD4(+) regulatory T cells through GITR breaks immunological self-tolerance. *Nat. Immunol.* **3**, 135-142 (2002).

30. Bacchetta, R. *et al.* Growth and expansion of human T regulatory type 1 cells are independent from TCR activation but require exogenous cytokines. *Eur. J. Immunol.* **32**, 2237-2245 (2002).

31. Walker, L.S. Treg and CTLA-4: two intertwining pathways to immune tolerance. *J. Autoimmun.* **45**, 49-57 (2013).

32. Sage, P.T., Paterson, A.M., Lovitch, S.B. & Sharpe, A.H. The coinhibitory receptor CTLA-4 controls B cell responses by modulating T follicular helper, T follicular regulatory, and T regulatory cells. *Immunity* **41**, 1026-1039 (2014).

33. Gorbachev, A.V. & Fairchild, R.L. CD4+CD25+ regulatory T cells utilize FasL as a mechanism to restrict DC priming functions in cutaneous immune responses. *Eur. J. Immunol.* **40**, 2006-2015 (2010).

34. Zhu, C. *et al.* An IL-27/NFIL3 signalling axis drives Tim-3 and IL-10 expression and T-cell dysfunction. *Nature communications* **6**, 6072 (2015).

35. Gupta, S. *et al.* Allograft rejection is restrained by short-lived TIM-3+PD-1+Foxp3+ Tregs. *J. Clin. Invest.* **122**, 2395-2404 (2012).

36. Zhu, S., Lin, J., Qiao, G., Wang, X. & Xu, Y. Tim-3 identifies exhausted follicular helper T cells in breast cancer patients. *Immunobiology* **221**, 986-993 (2016).

37. Huang, C.T. *et al.* Role of LAG-3 in regulatory T cells. *Immunity* **21**, 503-513 (2004).

38. Chen, X. *et al.* PD-1 regulates extrathymic regulatory T-cell differentiation. *Eur. J. Immunol.* **44**, 2603-2616 (2014).

39. Burton, B.R. *et al.* Sequential transcriptional changes dictate safe and effective antigen-specific immunotherapy. *Nature communications* **5**, 4741 (2014).

40. Yu, X. *et al.* The surface protein TIGIT suppresses T cell activation by promoting the generation of mature immunoregulatory dendritic cells. *Nat. Immunol.* **10**, 48-57 (2009).

41. Godefroy, E., Zhong, H., Pham, P., Friedman, D. & Yazdanbakhsh, K. TIGIT-positive circulating follicular helper T cells display robust B-cell help functions: potential role in sickle cell alloimmunization. *Haematologica* **100**, 1415-1425 (2015).

42. Collison, L.W. *et al.* The inhibitory cytokine IL-35 contributes to regulatory T-cell function. *Nature* **450**, 566-569 (2007).

43. Facciotti, F. *et al.* IL-10-producing forkhead box protein 3-negative regulatory T cells inhibit B-cell responses and are involved in systemic lupus erythematosus. *J. Allergy Clin. Immunol.* **137**, 318-321 e315 (2016).

44. Groux, H. *et al.* A CD4+ T-cell subset inhibits antigen-specific T-cell responses and prevents colitis. *Nature* **389**, 737-742 (1997).

45. Liu, H., Hu, B., Xu, D. & Liew, F.Y. CD4+CD25+ regulatory T cells cure murine colitis: the role of IL-10, TGF-beta, and CTLA4. *J. Immunol.* **171**, 5012-5017 (2003).

46. Zhu, Y., Zou, L. & Liu, Y.C. T follicular helper cells, T follicular regulatory cells and autoimmunity. *Int. Immunol.* **28**, 173-179 (2016).

47. Pot, C. *et al.* Cutting edge: IL-27 induces the transcription factor c-Maf, cytokine IL-21, and the costimulatory receptor ICOS that coordinately act together to promote differentiation of IL-10-producing Tr1 cells. *J. Immunol.* **183**, 797-801 (2009).

48. Chtanova, T. *et al.* T follicular helper cells express a distinctive transcriptional profile, reflecting their role as non-Th1/Th2 effector cells that provide help for B cells. *J. Immunol.* **173**, 68-78 (2004).

49. King, I.L. & Mohrs, M. IL-4-producing CD4+ T cells in reactive lymph nodes during helminth infection are T follicular helper cells. *J. Exp. Med.* **206**, 1001-1007 (2009).

50. Burzyn, D. *et al.* A special population of regulatory T cells potentiates muscle repair. *Cell* **155**, 1282-1295 (2013).

51. Brockmann, L. *et al.* Molecular and functional heterogeneity of IL-10-producing CD4(+) T cells. *Nature communications* **9**, 5457 (2018).

52. Diefenhardt, P. *et al.* IL-10 Receptor Signaling Empowers Regulatory T Cells to Control Th17 Responses and Protect from GN. *J. Am. Soc. Nephrol.* **29**, 1825-1837 (2018).

53. Zhao, Z. *et al.* IL-12R beta 2 promotes the development of CD4+CD25+ regulatory T cells. *J. Immunol.* **181**, 3870-3876 (2008).

54. Comes, A. *et al.* CD25+ regulatory T cell depletion augments immunotherapy of micrometastases by an IL-21-secreting cellular vaccine. *J. Immunol.* **176**, 1750-1758 (2006).

55. Meka, R.R., Venkatesha, S.H., Dudics, S., Acharya, B. & Moudgil, K.D. IL-27-induced modulation of autoimmunity and its therapeutic potential. *Autoimmun Rev* **14**, 1131-1141 (2015).

56. Villarino, A.V. *et al.* Positive and negative regulation of the IL-27 receptor during lymphoid cell activation. *J. Immunol.* **174**, 7684-7691 (2005).

57. Batten, M. *et al.* IL-27 supports germinal center function by enhancing IL-21 production and the function of T follicular helper cells. *J. Exp. Med.* **207**, 2895-2906 (2010).

58. Liu, W. *et al.* CD127 expression inversely correlates with FoxP3 and suppressive function of human CD4+ T reg cells. *J. Exp. Med.* **203**, 1701-1711 (2006).

59. McDonald, P.W. *et al.* IL-7 signalling represses Bcl-6 and the TFH gene program. *Nature communications* **7**, 10285 (2016).

60. Sakaguchi, S., Sakaguchi, N., Asano, M., Itoh, M. & Toda, M. Immunologic self-tolerance maintained by activated T cells expressing IL-2 receptor alpha-chains (CD25). Breakdown of a single mechanism of self-tolerance causes various autoimmune diseases. *J. Immunol.* **155**, 1151-1164 (1995).

61. Ballesteros-Tato, A. *et al.* Interleukin-2 inhibits germinal center formation by limiting T follicular helper cell differentiation. *Immunity* **36**, 847-856 (2012).

62. Liu, Y. *et al.* A critical function for TGF-beta signaling in the development of natural CD4+CD25+Foxp3+ regulatory T cells. *Nat. Immunol.* **9**, 632-640 (2008).

63. Ouyang, W., Beckett, O., Ma, Q. & Li, M.O. Transforming growth factor-beta signaling curbs thymic negative selection promoting regulatory T cell development. *Immunity* **32**, 642-653 (2010).

64. Apetoh, L. *et al.* The aryl hydrocarbon receptor interacts with c-Maf to promote the differentiation of type 1 regulatory T cells induced by IL-27. *Nat. Immunol.* **11**, 854-861 (2010).

65. Liu, X. *et al.* Transcription factor achaete-scute homologue 2 initiates follicular T-helper-cell development. *Nature* **507**, 513-518 (2014).

66. Edwards, C.L. *et al.* The Role of BACH2 in T Cells in Experimental Malaria Caused by Plasmodium chabaudi chabaudi AS. *Front. Immunol.* **9**, 2578 (2018).

67. Grant, F.M. *et al.* BACH2 drives quiescence and maintenance of resting Treg cells to promote homeostasis and cancer immunosuppression. *J. Exp. Med.* **217** (2020).

68. Lahmann, A. *et al.* Bach2 Controls T Follicular Helper Cells by Direct Repression of Bcl-6. *J. Immunol.* **202**, 2229-2239 (2019).

69. Karwacz, K. *et al.* Critical role of IRF1 and BATF in forming chromatin landscape during type 1 regulatory cell differentiation. *Nat. Immunol.* **18**, 412-421 (2017).

70. Ise, W. *et al.* The transcription factor BATF controls the global regulators of class-switch recombination in both B cells and T cells. *Nat. Immunol.* **12**, 536-543 (2011).

71. Nurieva, R.I. *et al.* Bcl6 mediates the development of T follicular helper cells. *Science* **325**, 1001-1005 (2009).

72. Tanaka, S. *et al.* CCAAT/enhancer-binding protein alpha negatively regulates IFN-gamma expression in T cells. *J. Immunol.* **193**, 6152-6160 (2014).

73. Bao, R. *et al.* Adenosine and the adenosine A2A receptor agonist, CGS21680, upregulate CD39 and CD73 expression through E2F-1 and CREB in regulatory T cells isolated from septic mice. *Int. J. Mol. Med.* **38**, 969-975 (2016).

74. Okamura, T. *et al.* CD4+CD25-LAG3+ regulatory T cells controlled by the transcription factor Egr-2. *Proc. Natl. Acad. Sci. U. S. A.* **106**, 13974-13979 (2009).

75. Zhang, P. *et al.* Eomesodermin promotes the development of type 1 regulatory T (TR1) cells. *Sci Immunol* **2** (2017).

76. Schaer, D.A. *et al.* GITR pathway activation abrogates tumor immune suppression through loss of regulatory T cell lineage stability. *Cancer Immunol Res* **1**, 320-331 (2013).

77. Wang, H. *et al.* The transcription factor Foxp1 is a critical negative regulator of the differentiation of follicular helper T cells. *Nat. Immunol.* **15**, 667-675 (2014).

78. Hori, S., Nomura, T. & Sakaguchi, S. Control of regulatory T cell development by the transcription factor Foxp3. *Science* **299**, 1057-1061 (2003).

79. Miyazaki, M. *et al.* Id2 and Id3 maintain the regulatory T cell pool to suppress inflammatory disease. *Nat. Immunol.* **15**, 767-776 (2014).

80. Choi, Y.S. *et al.* Bcl6 expressing follicular helper CD4 T cells are fate committed early and have the capacity to form memory. *J. Immunol.* **190**, 4014-4026 (2013).

81. Jin, J.O., Han, X. & Yu, Q. Interleukin-6 induces the generation of IL-10-producing Tr1 cells and suppresses autoimmune tissue inflammation. *J. Autoimmun.* **40**, 28-44 (2013).

82. Kwon, H. *et al.* Analysis of interleukin-21-induced Prdm1 gene regulation reveals functional cooperation of STAT3 and IRF4 transcription factors. *Immunity* **31**, 941-952 (2009).

83. Lee, J.Y. *et al.* The transcription factor KLF2 restrains CD4(+) T follicular helper cell differentiation. *Immunity* **42**, 252-264 (2015).

84. Choi, Y.S. *et al.* LEF-1 and TCF-1 orchestrate T(FH) differentiation by regulating differentiation circuits upstream of the transcriptional repressor Bcl6. *Nat. Immunol.* **16**, 980-990 (2015).

85. Ulges, A. *et al.* Protein kinase CK2 enables regulatory T cells to suppress excessive TH2 responses in vivo. *Nat. Immunol.* **16**, 267-275 (2015).

86. Bauquet, A.T. *et al.* The costimulatory molecule ICOS regulates the expression of c-Maf and IL-21 in the development of follicular T helper cells and TH-17 cells. *Nat. Immunol.* **10**, 167-175 (2009).

87. Dias, S. *et al.* Effector Regulatory T Cell Differentiation and Immune Homeostasis Depend on the Transcription Factor Myb. *Immunity* **46**, 78-91 (2017).

88. Angelin, A. *et al.* Foxp3 Reprograms T Cell Metabolism to Function in Low-Glucose, High-Lactate Environments. *Cell metabolism* **25**, 1282-1293 e1287 (2017).

89. Herold, M. *et al.* Liver X receptor activation promotes differentiation of regulatory T cells. *PLoS One* **12**, e0184985 (2017).

90. Heinemann, C. *et al.* IL-27 and IL-12 oppose pro-inflammatory IL-23 in CD4+ T cells by inducing Blimp1. *Nature communications* **5**, 3770 (2014).

91. Johnston, R.J. *et al.* Bcl6 and Blimp-1 are reciprocal and antagonistic regulators of T follicular helper cell differentiation. *Science* **325**, 1006-1010 (2009).

92. Fu, T. *et al.* Accelerated acute allograft rejection accompanied by enhanced T-cell proliferation and attenuated Treg function in RBP-J deficient mice. *Mol. Immunol.* **48**, 751-759 (2011).

93. Baumjohann, D. *et al.* The microRNA cluster miR-17 approximately 92 promotes TFH cell differentiation and represses subset-inappropriate gene expression. *Nat. Immunol.* **14**, 840-848 (2013).

94. Komatsu, N. *et al.* Pathogenic conversion of Foxp3+ T cells into TH17 cells in autoimmune arthritis. *Nat. Med.* **20**, 62-68 (2014).

95. Wang, H. *et al.* IL-27 induces the differentiation of Tr1-like cells from human naive CD4+ T cells via the phosphorylation of STAT1 and STAT3. *Immunol. Lett.* **136**, 21-28 (2011).

96. Choi, Y.S., Eto, D., Yang, J.A., Lao, C. & Crotty, S. Cutting edge: STAT1 is required for IL-6-mediated Bcl6 induction for early follicular helper cell differentiation. *J. Immunol.* **190**, 3049-3053 (2013).

97. Iwasaki, Y. *et al.* Egr-2 transcription factor is required for Blimp-1-mediated IL-10 production in IL-27-stimulated CD4+ T cells. *Eur. J. Immunol.* **43**, 1063-1073 (2013).

98. Koch, M.A. *et al.* T-bet(+) Treg cells undergo abortive Th1 cell differentiation due to impaired expression of IL-12 receptor beta2. *Immunity* **37**, 501-510 (2012).

99. Schmitt, N. *et al.* IL-12 receptor beta1 deficiency alters in vivo T follicular helper cell response in humans. *Blood* **121**, 3375-3385 (2013).

100. Ghoreishi, M. *et al.* Expansion of antigen-specific regulatory T cells with the topical vitamin d analog calcipotriol. *J. Immunol.* **182**, 6071-6078 (2009).

101. Papa, I. *et al.* TFH-derived dopamine accelerates productive synapses in germinal centres. *Nature* **547**, 318-323 (2017).

102. Grossman, W.J. *et al.* Differential expression of granzymes A and B in human cytotoxic lymphocyte subsets and T regulatory cells. *Blood* **104**, 2840-2848 (2004).

103. Cao, X. *et al.* Granzyme B and perforin are important for regulatory T cell-mediated suppression of tumor clearance. *Immunity* **27**, 635-646 (2007).

104. Chen, Z. *et al.* Ubiquitination signals critical to regulatory T cell development and function. *Int. Immunopharmacol.* **16**, 348-352 (2013).

105. Mandapathil, M. *et al.* Adenosine and prostaglandin E2 cooperate in the suppression of immune responses mediated by adaptive regulatory T cells. *J. Biol. Chem.* **285**, 27571-27580 (2010).

106. Deaglio, S. *et al.* Adenosine generation catalyzed by CD39 and CD73 expressed on regulatory T cells mediates immune suppression. *J. Exp. Med.* **204**, 1257-1265 (2007).

107. Huang, W., Solouki, S., Koylass, N., Zheng, S.G. & August, A. ITK signalling via the Ras/IRF4 pathway regulates the development and function of Tr1 cells. *Nature communications* **8**, 15871 (2017).

108. Huang, W., Jeong, A.R., Kannan, A.K., Huang, L. & August, A. IL-2-inducible T cell kinase tunes T regulatory cell development and is required for suppressive function. *J. Immunol.* **193**, 2267-2272 (2014).

109. Iyer, S.S. *et al.* Identification of novel markers for mouse CD4(+) T follicular helper cells. *Eur. J. Immunol.* **43**, 3219-3232 (2013).

110. Azzi, J. *et al.* Serine protease inhibitor 6 plays a critical role in protecting murine granzyme B-producing regulatory T cells. *J. Immunol.* **191**, 2319-2327 (2013).
